# A Randomized Controlled Trial Comparing the Effect of Phenylephrine by Intramuscular Route With Intravenous Infusion in Maintaining Haemodynamic Stability During Elective Lower Segment Caesarean Section Under Spinal Anaesthesia

**DOI:** 10.7759/cureus.34118

**Published:** 2023-01-23

**Authors:** Chandrekanth Lakshmikanthan, Balasubramaniam Gayathri, Swetha Ramani, Natarajan Balasubramanian

**Affiliations:** 1 Anaesthesiology, SRM Medical College Hospital and Research Centre (MCHRC), Chennai, IND

**Keywords:** intravenous infusion, intramuscular, phenylephrine, caesarean section, spinal anaesthesia, hypotension

## Abstract

Background

Hypotension is a commonly encountered side effect in patients undergoing spinal anaesthesia, particularly in patients undergoing caesarean section. Phenylephrine is a widely used drug to treat spinal-induced hypotension and to maintain hemodynamic stability. Our aim is to evaluate the effectiveness of phenylephrine given through two different routes prophylactically in prevention of post-spinal hypotension in patients undergoing caesarean section.

Methods

A total of 150 healthy pregnant women undergoing elective caesarean section were randomly allocated into three groups: Group M (prophylactic intramuscular use of 2 mg phenylephrine), group V (prophylactic intravenous infusion of 30 mcg phenylephrine per minute), and group P (no prophylaxis), rescue phenylephrine 30 mcg IV and atropine 0.6 mg IV were used intraoperatively to treat bradycardia and hypotension in all three groups. The primary outcome was maternal hemodynamic changes.

Results

There was an insignificant difference in demographic data between the groups. Maternal systolic and diastolic blood pressure were more stable in group M compared to group V and group P. Heart rate was significantly lower only in group V. We did not observe any statistical difference between the groups in the APGAR score or the fetal arterial blood gas values. The incidence of nausea and vomiting was more in group P.

Conclusion

Preventive intramuscular phenylephrine exhibited a more stable maternal hemodynamics when compared with the prophylactic intravenous infusion of phenylephrine and placebo in elective caesarean under spinal anaesthesia.

## Introduction

A well-known complication of spinal anesthesia for a caesarean section is hypotension due to the sympathetic blockade. Even a slight delay in treating hypotension might compromise the fetoplacental perfusion, leading to fetal acidosis and low APGAR scores. Spinal hypotension is usually treated by pre- or co-loading with intravenous fluids [1]. In patients exhibiting a significant fall in blood pressure and not responding to intravenous fluids, vasopressors such as ephedrine, phenylephrine or norepinephrine are used.

Multiple studies comparing prophylactic ephedrine and phenylephrine are available in published literature and have concluded that prophylactic phenylephrine is associated with better maternal and fetal outcomes [2,3]. However, most of these studies have been conducted outside India where the body composition is different due to ethnicity, lifestyle and body mass index. Those results cannot be extrapolated directly from the western population. Additionally, in these studies, the doses of prophylactic phenylephrine used to prevent spinal-induced hypotension have a more comprehensive prescription range in various routes. The authors do not explicitly recommend a correct and concrete amount of the drug or an injection route. We devised this study to compare phenylephrine's hemodynamic effects through intramuscular (IM) and intravenous (IV) infusion. We have preferentially used doses in the lower range provided in various studies, as a high dose of phenylephrine is known to cause bradycardia, which may result in reduced cardiac output in turn resulting in reduced uteroplacental flow which can be hazardous to the fetus [4]. Furthermore, we have included a third group where a bolus injection of phenylephrine was given whenever there was hypotension. To our knowledge, this is the first study where all three modes of phenylephrine with a relatively low dose for patients undergoing spinal anaesthesia are compared and evaluated for efficacy and complications.

## Materials and methods

This subject-blinded randomised controlled study was conducted in a tertiary care hospital in south India and it was approved by the local institutional ethics committee (IEC 2932/ IEC/2021) and registered at CTRI (CTRI/2022/06/043359). The study was designed according to the CONSORT 2010 checklist. The study aimed to find the efficacy and safety of prophylactic phenylephrine in preventing hypotension during spinal anaesthesia in patients undergoing caesarean section. The primary objective was the variation in maternal heart rate (HR) and blood pressure (BP) and the secondary objective was fetal acidosis as measured by neonatal umbilical artery pH. A total of 150 parturients undergoing an elective caesarean section from August 2021 to July 2022 were screened for enrolment. Those with a singleton pregnancy at term posted for elective caesarean section, aged from 18 to 40 years, American Society of Anesthesiologists (ASA) II grade and body mass index (BMI) less than 30 were included in the study. Parturients with multiple pregnancies, pregnancy-induced hypertension, preoperative history of bradycardia (heart rate (HR) less than 60/min), fetal malformation, fetal distress, any contraindications for spinal anaesthesia and those undergoing emergency caesarean sections were excluded from the study.

**Figure 1:**
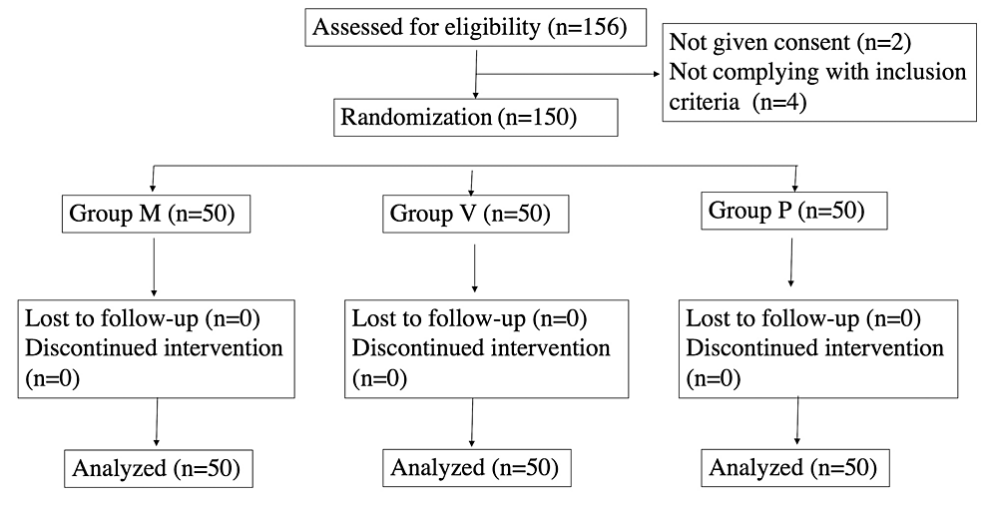
CONSORT diagram

After written informed consent patients were divided into three groups: group M with 50 patients receiving prophylaxis of 2 mg intramuscular (IM) injection of phenylephrine, group V with 50 patients receiving prophylaxis of intravenous (IV) infusion of phenylephrine at the rate of 30 mcg/min, and group P with 50 patients as controls receiving no prophylaxis. These proceedings are illustrated in Figure 1 below.

Random sequence was generated by computer-generated random numbers, and the patients were randomly assigned to the three groups. Allocation concealment was achieved by the sealed envelope technique. Demographic data including age, height and weight of the patients were documented, and HR, baseline systolic blood pressure (SBP) and diastolic blood pressure (DBP) were recorded. Spinal anaesthesia (SA) was administered in the left lateral position at L3-4 vertebral interspace with 10 mg heavy bupivacaine. No medications were given to supplement the effect of SA.

For patients in group M, 2 mg IM phenylephrine was administered 10 minutes before giving a subarachnoid block; for those in group V, IV infusion of phenylephrine at the rate of 30 mcg/min was started immediately after giving the subarachnoid block; and for group P, only intravenous fluids (RL) were administered at the 20 ml/kg/h. A bolus dose of 30 mcg of phenylephrine was administered whenever the systolic blood pressure fell below 90 mm Hg or 20% of baseline. In group V (IV infusion group), phenylephrine infusion was stopped after baby delivery. HR and BP were measured every 2 minutes for the first 10 minutes, every 5 minutes until 30 minutes, and every 15 minutes until 90 minutes for patients in all three groups.

Bradycardia was defined as HR < 50/min and was treated with 0.6 mg atropine. Hypertension was defined as SBP >20% of baseline. In group V, phenylephrine infusion was stopped if the SBP was higher than 20% of the baseline. In group M, if SBP was more than 140/80 mm Hg, NTG infusion was started (0.5 to 5 mcg/kg/h) and tapered to bring back the BP to normal range. Hypotension was defined as a fall in SPB >20% of baseline and treated with 30 mcg IV phenylephrine. After baby delivery, 1 and 5 minutes APGAR was noted. Umbilical blood was collected for arterial blood gas (ABG) analysis, and complications such as nausea, vomiting and shivering were recorded. After the surgery, the patients were shifted to the post-natal ward for continuous monitoring.

Using multiple comparisons of proportions, one-way test was conducted to evaluate whether each of the two treatment group proportions was significantly different from the control group at the 0.05 significance level. The actual powers for each test were 0.802 and 1.000, which were computed assuming that the actual control group proportion of hypotension is 0.120 and the treatment group’s 0.390 and 0.730, based on the work of Xu et al. [3]. A sample size of 45 in each group was obtained. Moreover, allowing 10% attrition, sample sizes of 50 in the control and individual treatment groups were finalized. Data were presented as mean, standard deviation, frequency and percentage. Continuable variables were compared using one-way ANOVA. Categorical variables were compared using Pearson chi-square test, and significance was defined by P values less than 0.05 using a two-tailed test. Data analysis was performed using IBM-SPSS version 21.0 (IBM Corp., Armonk, NY).

## Results

On comparing the demographic profiles, we found no significant differences between age, height, weight, body mass index (BMI) or American Society of Anesthesiologists (ASA) physical status of the patients in the three groups, as indicated in Table 1 below. The duration of surgery was around 50-60 min and comparable between the groups.

**Table 1:** Demographic variables ASA: American Society of Anesthesiologists

	Group M	Group V	Group P	p-value
Mean Age	26.76±3.48	26.48±3.54	26.98±3.82	0.787
Mean Height	155.22±4.72	154.42±4.73	156.08±4.91	0.220
Mean Weight	65.04±7.29	64.14±6.85	63.38±5.82	0.463
ASA Status	2	2	2	1

**Figure 2:**
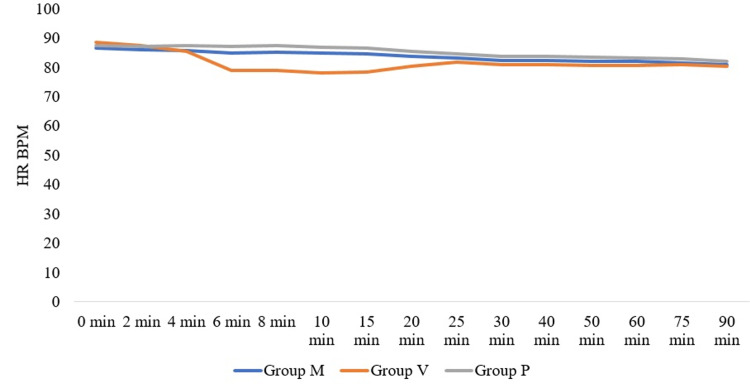
Distribution of HR HR: Heart  rate, BPM: Beats/minute

We observed a fall in heart rate (HR) in groups V (intravenous group) and M (intramuscular group), mainly between 6 to 15 minutes following spinal anaesthesia. The fall in heart rate (HR) was found to be more significant in group V when compared to group M. These details are presented in Figure 2.

**Figure 3:**
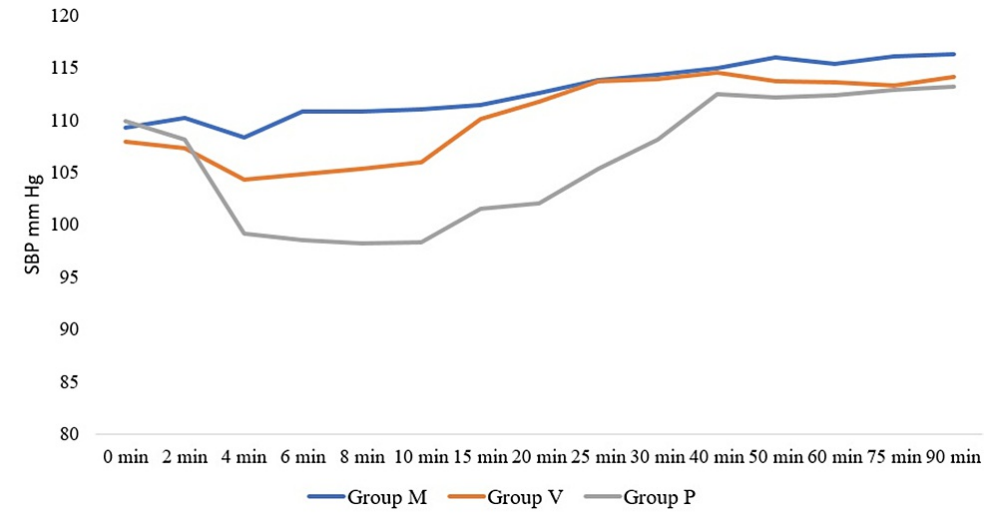
Distribution of systolic blood pressure SBP: Systolic Blood Pressure

**Figure 4:**
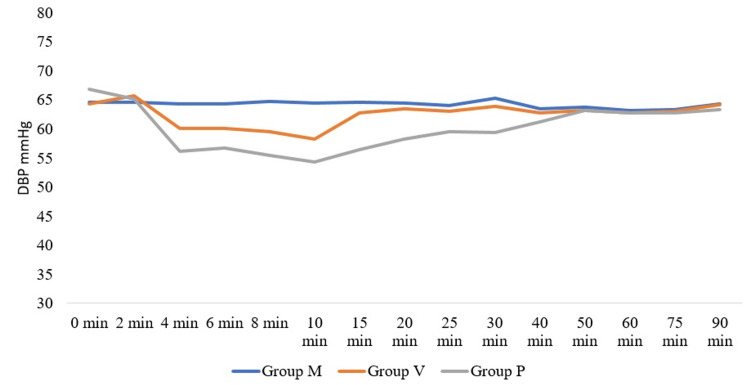
Distribution of diastolic blood pressure DBP: Diastolic Blood Pressure

Patients in group P did not exhibit a fall in HR. Two patients in the IV group required atropine. A total of 26 out of 50 patients in group P (control group) experienced a significant fall in both systolic and diastolic blood pressure from the 4th until 30th minute of spinal anaesthesia. No patient in group M had hypotension. Eight patients in group V receiving IV phenylephrine exhibited a significant fall in systolic blood pressure (SBP) and diastolic blood pressure (DBP) from 4th until 10th minute. These data are illustrated in Figure 3 and Figure 4 below.

Rescue phenylephrine was required by 26/50 patients in the control group and 8/50 patients in the IV group. The time rescue phenylephrine was given much earlier in group P (control group) - 5.12 minutes; compared to 6.38 minutes in group V (intravenous group). In group M (intramuscular group), no hypotensive episode was recorded. The umbilical artery blood gas and 5 min APGAR were reasonable and comparable among the groups. These data are presented in detail in Table 2.

**Table 2:** Data representing various secondary objectives PaO2 - Partial pressure of oxygen; PaCO2 - Partial pressure of carbon dioxide; NA - Not applicable.

	Group M	Group V	Group P	p-value
Umbilical artery pH	7.31±0.05	7.28±0.05	7.29±0.05	0.190
Umbilical artery PaO2	24.7±5.94	24.4±6.50	23±5.97	0.339
Umbilical artery PaCO2	39.16±3.23	39.49±3.44	38.34±2.97	0.201
APGAR score at 1 minute >7	50	50	50	1
APGAR score at 5 minutes >8	50	50	50	1
Time to the first use of rescue phenylephrine after anaesthesia	N.A.	6.38±1.58	5.12±2.07	<0.0001
Total number of patients requiring rescue phenylephrine doses	0	8	26	<0.0001
Total number of patients requiring rescue atropine during surgery	0	2	0	0.132
Incidence of nausea	0	3	10	0.001
Incidence of vomiting	0	1	5	0.26

Nausea and vomiting were not observed in group M. Nausea was experienced by 10/50 patients in group P and 3/50 patients in group V. Vomiting was experienced by 5/50 patients in group P and 1/50 patients in group V.

## Discussion

Hypotension is a well-known and inevitable complication of spinal anaesthesia in pregnant women undergoing a caesarean section. It affects the mother and the fetus, with potential complications such as fetal acidosis and reduced uteroplacental perfusion. Many drugs have been advocated for treating and preventing hypotension, including ephedrine, noradrenaline and dopamine. However, phenylephrine is the most popular and widely used. This study aims to compare two routes of prophylactic phenylephrine - 2 mg intramuscular (IM) and 30 mcg/min intravenous (IV) infusion, with a third control group in which phenylephrine was administered as a single bolus of 30 mcg whenever BP fell to <90 mmHg.

We observed a more significant fall in heart rate (HR) in group V in which patients received prophylactic IV infusion of 30 mcg/min phenylephrine when compared to group M where patients received 2 mg of prophylactic intramuscular phenylephrine. Those in the placebo group who received 30 mcg IV rescue bolus of phenylephrine did not exhibit a fall in HR. Phenylephrine is an α-receptor agonist without β-agonist activity and its administration causes an increase in arterial blood pressure and a decrease in heart rate due to vasoconstriction and baroreceptor activation, respectively. Although the patients in groups M and P had bradycardia, two individuals in the IV group had HR <50, thus requiring atropine. In both cases, patients had SBP >140 mm Hg, and phenylephrine infusion was stopped. No further episodes of bradycardia were noticed. Bradycardia warranting the administration of Atropine was not noticed in patients in groups M and P. In a study conducted by Xu et al. [3], comparing 5 mg prophylactic IM phenylephrine with prophylactic single dose of 100 mcg IV phenylephrine, 6% of patients receiving IM phenylephrine, 9% of patients in prophylactic IV phenylephrine and 12% patients in the placebo group had bradycardia [3].

We observed that patients in the IM group did not experience hypotensive episodes. Patients in the control group had a higher incidence of hypotension than the prophylactic IV group, thus requiring phenylephrine bolus doses. We attribute this to the delay in maintaining serum phenylephrine in the IV group, where infusion was started after SA. In contrast, in the IM group, the intramuscular injection was given before SA. In their study, Xu et al. [3] observed that 5 mg IM phenylephrine exhibited a more stable haemodynamic status when compared to prophylactic IV bolus group and control. Ayorinde et al. [4] compared different doses of IM phenylephrine and demonstrated a higher incidence of hypotension (70%) in the 2 mg IM phenylephrine group when compared to the patients treated with 4 mg of IM phenylephrine (33%). On the contrary, we noticed that a 2 mg IM dose was sufficient to prevent hypotension in our patients. Ethnicity and body composition might cause these differences, indicating that the results of Ayorinde et al. cannot be directly extrapolated to the Indian population. Allen et al. [5] compared different doses of intravenous phenylephrine. They observed that prophylactic fixed-rate intravenous infusions of 25 and 50 mcg of phenylephrine provided greater maternal hemodynamic stability than infusions of 75 and 100 mcg/min. Reactive maternal hypertension was observed in patients who were given phenylephrine IV infusion at 75 and 100 mcg per minute. The study concluded that a lower dose of phenylephrine would be more appropriate for fixed-dose prophylactic infusion to prevent maternal hypotension [5]. In our study, we used only 30 mcg/min phenylephrine IV infusion, and did not observe any reactive hypertension in any group. In a meta-analysis conducted by Heesen et al. [6], it was shown that the relative risk (95% CI) of hypotension with phenylephrine infusion was lower (0.36(0.18-0.73)) when compared to placebo (0.58(0.39-0.88)). However, this analysis did not conclude the ideal dose of phenylephrine at which spinal-induced hypotension can be treated or prevented [6]. In a study conducted by Moslemi and Rasooli [7] on women who underwent spinal anaesthesia for elective caesarean sections, systolic and diastolic blood pressures were best maintained with a prophylactic infusion of phenylephrine (450 mcg in 250 cc of normal saline over 30 minutes (15 mcg/min)) when compared to placebo (250 cc of normal saline infusion) [7]. We used prophylactic 30 mcg/min infusion and found it to maintain BP in the majority (42/50; 84%) of patients. In a study by das Neves et al., the infusion dose of 0.15 mcg/kg/min was found to be beneficial compared to a single dose of 50 mcg of phenylephrine received after initiation of spinal anaesthesia [8].

The pH of the umbilical artery blood gives us a fair idea about the acid-base status of the fetus. The mean pH levels noticed in our patients were 7.31 in group M, 7.28 in group V and 7.29 in group P; an umbilical artery pH lower than 7.2 is considered fetal acidosis [9-11]. In our study, no significant differences in the umbilical artery pH were noted among the groups, indicating that no patient had significant uncorrected hypotension. Phenylephrine is observed to significantly increase the perfusion index of uterine and placental arcuate arteries, thus effectively increasing maternal blood pressure without compromising fetal circulation [12-14]. A higher incidence of vomiting was noticed in patients in the placebo group, followed by the intravenous group. Patients receiving intramuscular phenylephrine did not have nausea or vomiting. The sympathetic block effect of spinal anaesthesia and the subsequent increase in parasympathetic activity could have led to nausea and vomiting via gastrointestinal hyperactivity in these patients [15].

Limitation

The uterine artery perfusion index is an effective modality to assess the fetal circulation during any intervention. Unfortunately, it was not used in our study due to the unavailability of resources. The study of uterine artery perfusion index could have given further insight into the dynamics of circulation and changes induced by phenylephrine. We limited the study to healthy pregnant women. Consequently, the results of this study cannot be extrapolated to pregnant women with multiple comorbidities, which warrants further trials.

## Conclusions

We conclude that the prophylactic 2 mg intramuscular phenylephrine provides more hemodynamic stability compared to prophylactic 30 mcg/min phenylephrine infusion. No complications like nausea and vomiting were observed. The neonatal APGAR and umbilical artery pH were good in all the groups.
